# Unexpected formation of *N*′-phenyl-thiophosphorohydrazidic acid *O*,*S*-dimethyl ester from acephate: chemical, biotechnical and computational study

**DOI:** 10.1007/s13205-015-0313-6

**Published:** 2015-12-23

**Authors:** Vijay Kumar, Sukhmanpreet Kaur, Simranjeet Singh, Niraj Upadhyay

**Affiliations:** 1 Department of Chemistry, Lovely Professional University, Punjab, 144411 India; 2 Department of Biotechnology, Lovely Professional University, Punjab, India; 3 Department of Chemistry, Dr. Harisingh Gour University, Sagar, Madhya Pradesh India; 4 Regional Pesticides Testing Laboratory, Ministry of Agriculture, Government of India, Chandigarh, 160030 India

**Keywords:** Acephate, Phenylhydrazine, Thermal analysis, Plant growth-promoting traits

## Abstract

By the nucleophilic attack of phenylhydrazine on acephate in aqueous medium, a stable product *N*′-phenyl-thiophosphorohydrazidic acid *O*,*S*-dimethyl ester (**1**) was obtained and characterized by elemental, spectral and thermal analysis. Thermodynamic parameters, Ea, Δ*H*°, Δ*S*° and Δ*G*°, have found to be 62.15, −67.95, −0.068 and −20.05 kJ mol^−1^ according to the Coats–Redfern equation. Analysis of interaction of **1** with BSA protein was done by using the UV–Vis and FTIR spectroscopic methods. The observed binding constants was 1.12 (±0.09) × 10^4^ M^−1^. The biological effect of **1** was checked on different plant growth-promoting (PGPR) strains such as *Rhizobium* *leguminosarum*, *Pseudomonas* *fluorescens*, *Arthrobacter* *citreus*, *Bacillus* *brevis* and *Salmonella* *typhimurium* and compared with parent molecule acephate where **1** has shown less toxicity against PGPRs as compared to acephate. The experimental results for geometric parameters and values of peak position in IR spectra have found to match excellently with the computational studies performed by GAMESS software package. Theoretically, twelve new analogs of **1** were prepared and their comparative reactivity (HOMO–LUMO energy) and biodecomposition (on the basis of polarizability) is discussed.

**Electronic supplementary material:**

The online version of this article (doi:10.1007/s13205-015-0313-6) contains supplementary material, which is available to authorized users.

## Introduction

All the contaminants including insecticides, nematodes and herbicides applied to crops reach to soil and influence the soil fertility by inhibiting the soil’s microorganisms. Moreover, plant protection has become necessary in order to increase the food production, and therefore, multiple and over use of pesticides are called necessary evil (David 1998; Krämer 2007; Prasad et al. 2013; Wasim et al. 2009). Pesticides are considered to be an integral part of modern agriculture. So the topics such as green decomposition, production of less toxic and green pesticides are the prime most requirements of current era.

Acephate is an important and cheapest organophosphorus insecticide used worldwide (Kumar et al. 2015a). In last 5 years, every year 10 % increase in production of technical grade acephate was observed (Kumar et al. 2015a). It was registered to control a wide range of insects on various agricultural crops. The insecticidal potency of acephate is due to the inhibition of acetyl-cholinesterase (AChE) activity (Kumar et al. 2013a, 2015a). It is toxic in various components of the environment and further decomposed into highly toxic methamidophos (Kumar et al. 2015a). Hence, the safe decomposition and preparation of less toxic analog of acephate are an important issue from the environmental concern.

Moreover, phenylhydrazine is classified among the aromatic nucleophile. It acts as an intermediate of various active compounds (Chen et al. 2014; Rosamilia et al. 2008). As per our best information, there is no reported data about the interactions of phenylhydrazine with acephate. Accordingly, the present study (in vitro) was initiated to investigate the ability of phenylhydrazine to decompose/neutralize the toxic effect of acephate and synthesis of green pesticide.

## Experimental

### Materials

Acephate technical grade (>90 %) was supplied by Gautmi Ltd., Andhra Pradesh, India. All the materials and solvents employed in synthesis were of extra-pure grade and used after further purification. Pure cultures of the strains, namely *Rhizobium* *leguminosarum* (NCIM-2749), *Pseudomonas* *fluorescens* (NCIM-5096), *Arthrobacter* *citreus* (NCIM-2320), *Bacillus* *brevis* (NCIM-2532) and *Salmonella* *typhimurium* (NCIM-2501), were purchased from National Chemical Laboratory, Pune, India.

### Chemistry

#### Decomposition/synthesis procedure

An environment-friendly one-step method was designed and followed where equimolar aqueous solution of acephate (0.018 g in 10 mL/0.01 M) mixed with one molar aqueous solution of phenylhydrazine (0.011 g in 10 mL/0.01 M) in a beaker. Solution of the beaker was stirred for 1 h at room temperature (RT) at pH 3 and kept for 7 days in the presence of normal light at RT and pH 3 (maintained by using HCl). Fine amorphous white-colored product (**1**) was obtained, which was washed with hot water, methanol and dried overnight at 45 °C and allowed for spectral, thermal, BSA binding and plant growth-promoting activities.

### Biology

#### Plant growth-promoting activities

As mentioned above, entire pollutants including insecticides, nematodes and herbicides applied to crops reach to soil and influence soil fertility by inhibiting the soil’s microorganisms. Hence, the study of the effect of newly synthesized molecule was seen on PGPR strains. In the current study, the plant growth-promoting activities, namely siderophore production, indole acetic acid production, hydrogen cyanide production and phosphate solubilization, were checked with above-mentioned strains (Kumar et al. 2015b). All the activities were preformed in triplicates, and results were analyzed by using the ANOVA statistical analysis.

#### Preparation of stock solution and interaction of **1** with BSA

Bovine serum albumin (40 mg/mL or 0.5 mM) was dissolved in aqueous solution containing phosphate buffer (pH 7.2). The protein concentration was determined spectrophotometrically using an extinction coefficient of 36,500 M^−1^ cm^−1^ at 275 nm (Painter et al. 1998). Solutions of **1** were first prepared in phosphate buffer/ethanol (50 %) and then diluted by serial dilution to 0.010, 0.0075, 0.0050, 0.0025, 0.00125 mM in the same phosphate buffer.

The absorption spectra were recorded on a Shimadzu 1800S double-beam spectrophotometer, using a slit of 5 nm; quartz cuvettes of 1 cm and scan speed of 250 nm min^−1^ were used. The UV–Vis absorptions of BSA in the presence and absence of **1** were measured at pH 7.2 by keeping the concentration of BSA constant (0.05 mM), while varying the concentration of **1** (0.010, 0.0075, 0.0050, 0.0025, 0.00125 mM), in the range of 230–400 nm. The binding constants of the drugs–BSA complexes were calculated as reported (mathematical detail is stated in Supplementary Information S3) (Abdi et al. 2012; Connors 1987).

#### FTIR spectroscopy measurements

Infrared measurement was taken at room temperature on a Shimadzu 8400S FTIR spectrometer, equipped with KBr beam splitter. FTIR study was performed as described by Abdi et al. (2012). Solution of **1** was added dropwise to the protein solution with constant stirring to ensure the formation of homogeneous solution. Interferograms were accumulated over the spectral range 4000–400 cm^−1^ with a nominal resolution of 4 cm^−1^ and 50 scans. At first, spectra of buffer (phosphate pH 7.4) and protein solution were collected on the same conditions. Then, buffer spectrum is subtracted from the spectra of sample solution to get the FTIR spectra of protein. The difference spectra [(protein solution + molecule solution) + (protein solution)] were generated using the polypeptide antisymmetric and symmetric C–H stretching bands located at 2900–2800 cm^−1^, as internal standard. These bands, which are due to protein C–H stretching vibrations, do not undergo any spectral changes (shifting or intensity variation) upon any interaction with drug, and therefore, they are commonly used as internal standard. When producing difference spectra, these bands were adjusted to the baseline level, in order to normalize difference spectra.

### Computational study

The theoretical, geometric parameters (bond lengths and bond angles, Huckel charge densities), polarizability, HOMO–LUMO energy difference and infrared intensities of the product in the ground state were calculated using HF method with the 6-311++G(d,p) basis set for the first time. Entire analysis was performed by GAMESS program package (Aihara 1999; Kumar et al. 2015c, d). The motive of the computational study was to assist and cross-check the experimental study and vice versa. Additional motive was to obtain the data such as steric energy, chemical reactivity, biodecomposition, Huckel charge densities, bond angles and bond length which are not experimentally obtained.

## Results and discussions

### Chemistry

#### Structure illustration

**Fig. 1:**
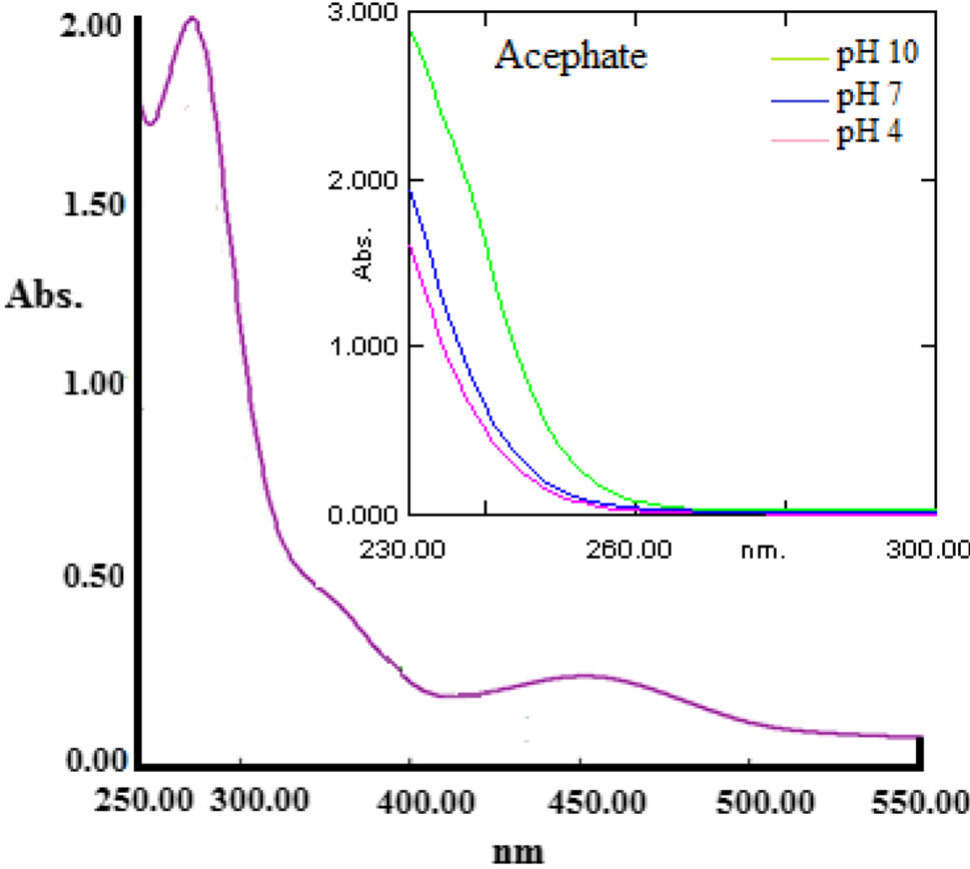
UV–Vis spectra of **1** and of acephate

The observed values of absorption maxima (*λ*_max_) for **1** in UV–Vis spectra were 269 nm (due to n → π* transitions) and 456 nm (due to π → π* transitions). By comparing the UV–Vis spectra of acephate with product (Fig. 1), it was observed that **1** contains an organic chromophoric group that leads to increase in wavelength as well as absorption. Comparatively, no sharp absorption maxima (*λ*_max_) were observed in the case of acephate even in UV region. Here, UV–Vis spectra clearly indicate the formation of product due to bond formation of phenylhydrazine with acephate.

**Fig. 2:**
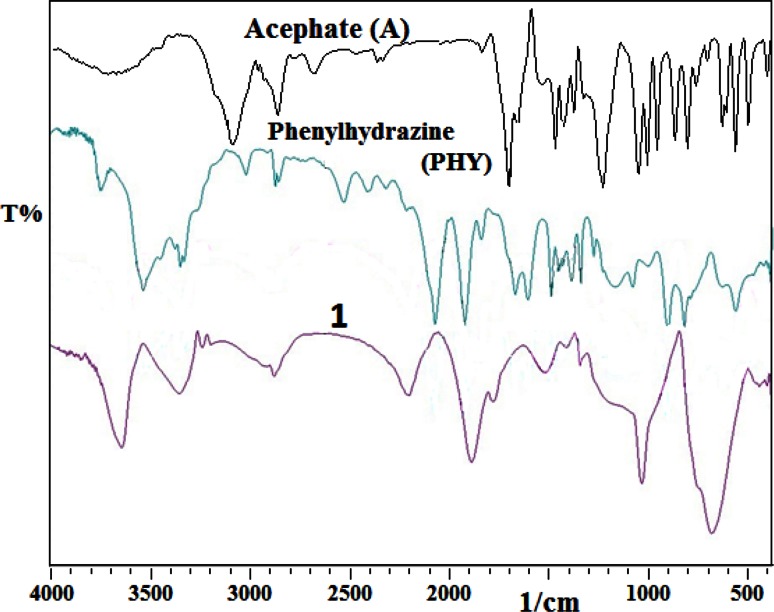
FTIR spectrum of acephate, phenylhydrazine and **1**

On comparison of IR spectra of acephate (IR spectra) and phenylhydrazine to **1**, new peak was observed at 3650 cm^−1^ that corresponds to stretching frequencies of free O–H of water. Broadening and shifting were observed in the stretching frequencies of N–H (3500–3300 cm^−1^) of phenylhydrazine and acephate. A new peak at 2190 cm^−1^ was observed due to stretching vibration of C=N=N type allene system. Redshift in the stretching band of C=N group of **1** (1630 cm^−1^) compared to phenylhydrazine (1900 cm^−1^) was observed. The *ν*(C=O) stretching band of acephate disappears in **1** that indicates removal of carbonyl group from acephate on attack of phenylhydrazine for the formation of **1**. Broad peak at 1500 cm^−1^ in **1** belongs to the stretching frequencies of C=C. Association of P=O oxygen with hydrogen atom of water molecule was observed in terms of broadening and increase in intensity of P–O stretching band (850 cm^−1^) (Fig. 2). The frequency for N–H out of plane was observed at 700 cm^−1^ in broad and intense form (Kumar et al. 2013a, b; Silberstein et al. 2005).

**Fig. 3:**
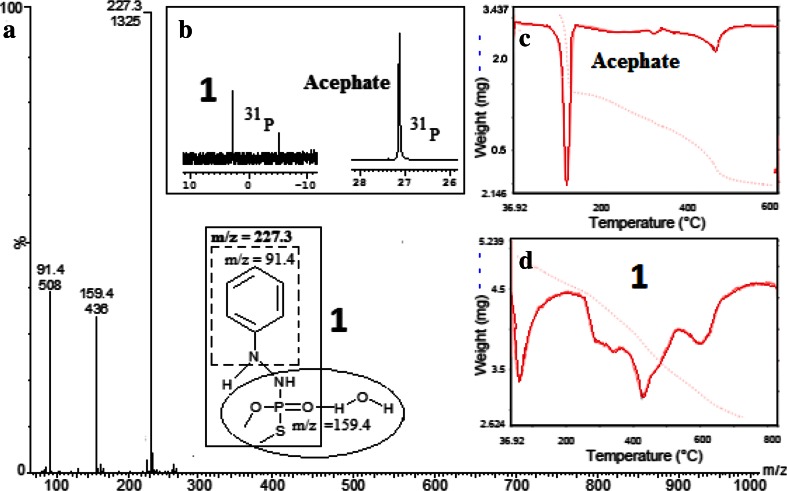
Mass (ESI–MS), ^31^P-NMR and thermal study (TGA) of acephate and **1**. **a** Mass analysis of **1**, **b** ^31^P NMR spectra of **1** and of acephate, **c** thermal study of acephate and **d** thermal study of **1**

Compound **1** displayed molecular ion peak at *m*/*z* 227.3 with relative abundance of 100 %, which is in excellent agreement with the structure shown in Fig. 3a. Hundred percentage intensity at 227.3 confirms the formation of a stable moiety *N*′-phenyl-thiophosphorohydrazidic acid *O*,*S*-dimethyl ester. Some other observed fragments with approximate intensity were at *m*/*z* = 159.4 (35 %) and 91.4 (40 %), respectively. In the fragmentation of **1**, no peak was observed at around *m*/*z* = 141, indicating that decomposition of **1** to methamidophos is improbable. ^31^P-NMR signals at 27.13 ppm were assigned to the P–O–CH_3_ and P–S–CH_3_ groups of acephate. Upward shift at 2.88 and −5.88 ppm was observed in **1** as compared to acephate due to two different arrangements of –S–CH_3_ and –O–CH_3_ around phosphate atoms (Kumar et al. 2013a; Silberstein et al. 2005). It means both the signal showed downshift (10 and 12 times) with decrease in intensity (2 and 3 times) as compared to acephate (Fig. 3b).

The presence of water molecules in **1** was confirmed by the thermal study. Higher activation energy for thermal decomposition of **1** was observed (62.15 kJ/mol) as compared to acephate (9.54 kJ/mol) (Fig. 3c, d; Table 1). Thermal analysis (TGA) showed formation of stable new adduct as compared to parent acephate. In thermal analysis, it was observed that product **1** decomposed at higher temperature (698 K) (Table 1) as compared to acephate (450 K).

**Table 1:** Kinetic parameters of the acephate and **1** (for detail, see Supplementary Information S4)

Moiety	Main step	Thermodynamic parameters by TG/DTG analysis						
		*T*_p_ (K)	*A* (min^−1^)	Ea (kJ/mol)	Δ*H* (kJ/mol)	Δ*S* (kJ/mol)	Δ*G* (kJ/mol)	*r* ^2^
Acephate **1**	1st	450	9.1 × 10^19^	9.54	5.79	0.167	−69.36	0.98
	2nd	698	6.3 × 10^7^	62.15	−67.95	−0.068	−20.04	0.96

#### Mechanism

**Scheme 1:**
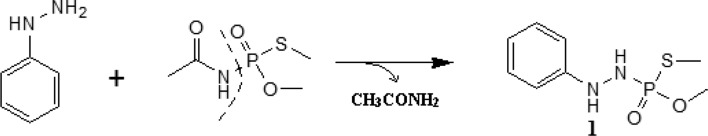
Synthesis scheme of **1**

The final structure of **1** was confirmed by FTIR, ^31^P-NMR, mass and thermal analysis data as sated above. Nucleophilic substitution reaction by attack of nucleophile phenylhydrazine (:NH_2_–NH–) to acephate is responsible for the formation of product **1**. Both phosphate phosphorus and carbonyl carbon are electron deficient but because of more electropositive character of phosphorus, nucleophile attack on it leads to displacement of acetamide (Chen et al. 2014; Rosamilia et al. 2008) (Scheme 1).

### Biology

#### Plant growth-promoting activities

Effect of **1** on plant growth-promoting strains was performed to check its toxic/negative effect compared to acephate at their different concentration levels, i.e., 25, 50, 100 and 200 µg/L. Effect on inhibition of siderophores production by acephate and **1** was determined on King’s B (KB) medium using the literature reported method (Yasmin et al. 2009). Huge inhibition difference was obtained between acephate (more toxic) and **1** (less toxic) (Ahemada and Kibret 2014). The siderophore production was reduced significantly (*p* ≤ 0.05) in acephate compared to **1** (and is shown by notation acephate: **1**). At 25 µg/L, inhibition of plant growth-promoting strain compared to control was found by 30:17 % and 80:55 % over control was obtained at 200 µg/L (Table 2). Exact siderophore production by different bacteria in the presence of **1** was found in order: *B.* *brevis* > *R.* *leguminosarum* > *S.* *typhimurium* > *A.* *citreus* = *P.* *fluorescens* (supplementary Figure S2).

**Table 2:** Plant growth-promoting activities of acephate and **1**

Strain code	Conc. (ppm)	Acephate				Molecule **1**			
		SA (mean)^a^	IAA (mean)^a^	HCN	PS (mm)^b^	SA (mean)^a^	IAA (mean)^a^	HCN	PS (mm)^b^
1	0	0.81 ± 0.006*	0.74 ± 0.007*	+++	12 ± 2*	0.81 ± 0.006*	0.74 ± 0.004*	+++	12 ± 2*
	25	0.56 ± 0.004*	0.54 ± 0.006*	+	08 ± 0*	0.75 ± 0.004*	0.64 ± 0.006*	++	10 ± 2*
	200	0.14 ± 0.004*	0.42 ± 0.006*	−	05 ± 1*	0.45 ± 0.003*	0.53 ± 0.007*	+	09 ± 1*
2	0	0.75 ± 0.005*	0.72 ± 0.005*	++	11 ± 2*	0.75 ± 0.002*	0.72 ± 0.005*	++	11 ± 3*
	25	0.58 ± 0.007*	0.53 ± 0.004*	+	07 ± 1*	0.67 ± 0.004*	0.58 ± 0.005*	++	09 ± 2*
	200	0.21 ± 0.007*	0.44 ± 0.006*	−	05 ± 1*	0.49 ± 0.006*	0.49 ± 0.003*	+	08 ± 2*
3	0	0.89 ± 0.006*	0.69 ± 0.007*	+++	14 ± 0*	0.89 ± 0.005*	0.69 ± 0.006*	+++	14 ± 2*
	25	0.61 ± 0.006*	0.59 ± 0.008*	+	09 ± 2*	0.69 ± 0.005*	0.62 ± 0.005*	++	11 ± 1*
	200	0.24 ± 0.008*	0.39 ± 0.008*	+	06 ± 0*	0.44 ± 0.007*	0.58 ± 0.005*	+	09 ± 2*
4	0	0.84 ± 0.006*	0.71 ± 0.007*	++	15 ± 3*	0.84 ± 0.003*	0.71 ± 0.008*	++	15 ± 4*
	25	0.68 ± 0.005*	0.64 ± 0.006*	+	11 ± 2*	0.76 ± 0.006*	0.67 ± 0.006*	++	12 ± 2*
	200	0.19 ± 0.006*	0.51 ± 0.005*	−	08 ± 2*	0.59 ± 0.004*	0.61 ± 0.007*	+	09 ± 1*
5	0	0.86 ± 0.005*	0.73 ± 0.006*	+++	12 ± 3*	0.86 ± 0.007*	0.73 ± 0.004*	+++	12 ± 3*
	25	0.63 ± 0.004*	0.59 ± 0.005*	+	09 ± 1*	0.69 ± 0.005*	0.65 ± 0.005*	++	10 ± 1*
	200	0.21 ± 0.008*	0.51 ± 0.005*	−	06 ± 0*	0.51 ± 0.006*	0.56 ± 0.006*	+	08 ± 0*

1, *Rhizobium* *leguminosarum*; 2, *Arthrobacter* *citreus*; 3, *Pseudomonas* *fluorescens*; 4, *Bacillus* *brevis*; 5, *Salmonella* *typhimurium*; SA, siderophoric activities; IAA, indole acetic acid activities; HCN hydrogen cyanide production; PS, phosphate solubilization; +++, stand for deep brown color; ++, stand for light brown; −, stand for no color

* Statistically significant (*p* ≤ 0.05) versus control

^a^Mean measured by comparing absorbance

^b^Mean measured by scale due to hole formation

Phosphate solubilization was increased with the use of **1** as compared with acephate (Table 2). Among all strains at significant level (*p* ≤ 0.05), *B.* *brevis* produced less phosphate solubilization (hole in mm) in the presence of **1.** Exact order of phosphate solubilization was found to be *R.* *leguminosarum* > *P.* *fluorescens* > *A.* *citreus* > *S.* *typhimurium* > *B.* *brevis* (supplementary Figure S3).

Rhizobacteria produce HCN to protect the growing plants from pathogen attack by direct killing of parasites (Ahemada and Kibret 2014). In this study, three concentrations of each acephate and **1** did affect negatively HCN synthesis, but very low effect of **1** was analyzed. In case of acephate, only two strains (*A.* *citreus*, *B.* *brevis*) were found to produce HCN (supplementary Fig. S4), while in the presence of **1** all strains (*R.* *leguminosarum*, *P.* *fluorescens*, *A.* *citreus*, *B.* *brevis* and *S.* *typhimurium*) produced HCN (Table 2).

In the medium, where acephate and **1** was not supplied, all strains produced indole acetic acid (IAA) in significant amount (*p* ≤ 0.05). But, on comparison with acephate, **1** was producing higher concentration of IAA (Table 2). Among all strains at significant level (*p* ≤ 0.05), *S.* *typhimurium* produced lowest amount of IAA in the presence of **1** compared to other strains and order of IAA production was: *A.* *citreus* > *B.* *brevis* > *R.* *leguminosarum* > *P.* *fluorescens* > *S.* *typhimurium*. It is worthy to mention that acephate shows inhibitory effect on plant growth-promoting strains even at small concentrations. It could disrupt the bacterial community due to differences in sensitivity between microorganisms. Any modification of the environment which leads to a response by living organisms may be considered as a stress (Kumar et al. 2015a; Missous et al. 2007). The biotic stress observed in biology, is considered a global phenomenon, and can be extended to anthropogenic pressure such as genetic engineering or xenobiotic (including pesticides) pollution (Kumar et al. 2014; Thammavongs et al. 2008). The synthesized molecule **1** did not show significant effect on the growth of the tested bacteria w.r.t. their plant growth-promoting activities. More detail of plant growth activities by **1** can be seen in Supplementary Information S5.

#### UV–Vis spectra and stability of **1**-BSA complexes

**Fig. 4:**
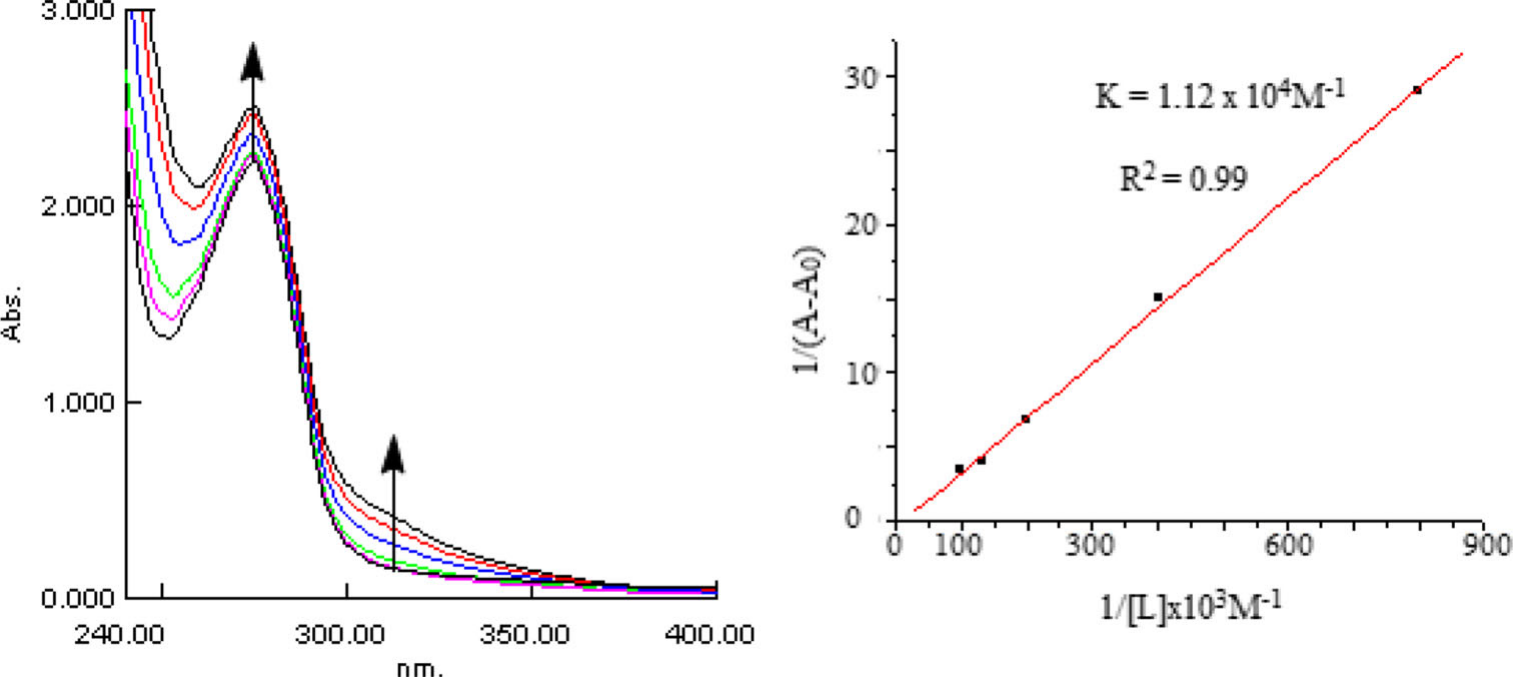
UV–Vis study of **1**-BSA binding constant

An increase in the concentrations of **1** in the presence of BSA resulted increase in UV light absorption and shifting of BSA band at 279–274 nm that can be related to complex formation (Fig. 4). Molecule showed binding with BSA with a binding constants of 1.12 (±0.09) × 10^4^ M^−1^. The binding constant for **1**-BSA complex suggests a low affinity (<10^6^) for complex formation, compared to strong ligand–protein complexes, with binding constants ranging from 10^6^ to 10^8^ M^−1^ (Bourassa et al. 2011; Kragh-Hansen 1990; Liu et al. 2004). In in vitro studies, organophosphate pesticides were not only found to bind with serine hydrolases but also to proteins that was not having serine as active site, e.g., Tyr 411 of human albumin and Lys 296 of mouse transferrin (Lockridge 2013). Higher the protein binding constant of agrochemicals or pesticides, higher will be the toxicity (Lockridge 2013; Kumar et al. 2013a). Interestingly, the low binding constant of **1** shows less toxicity of synthesized molecule toward protein interactions of organisms which is a major problem of maximum agrochemical especially in aquatic environment.

#### FTIR spectra of BSA and product-BSA complexes

**Fig. 5:**
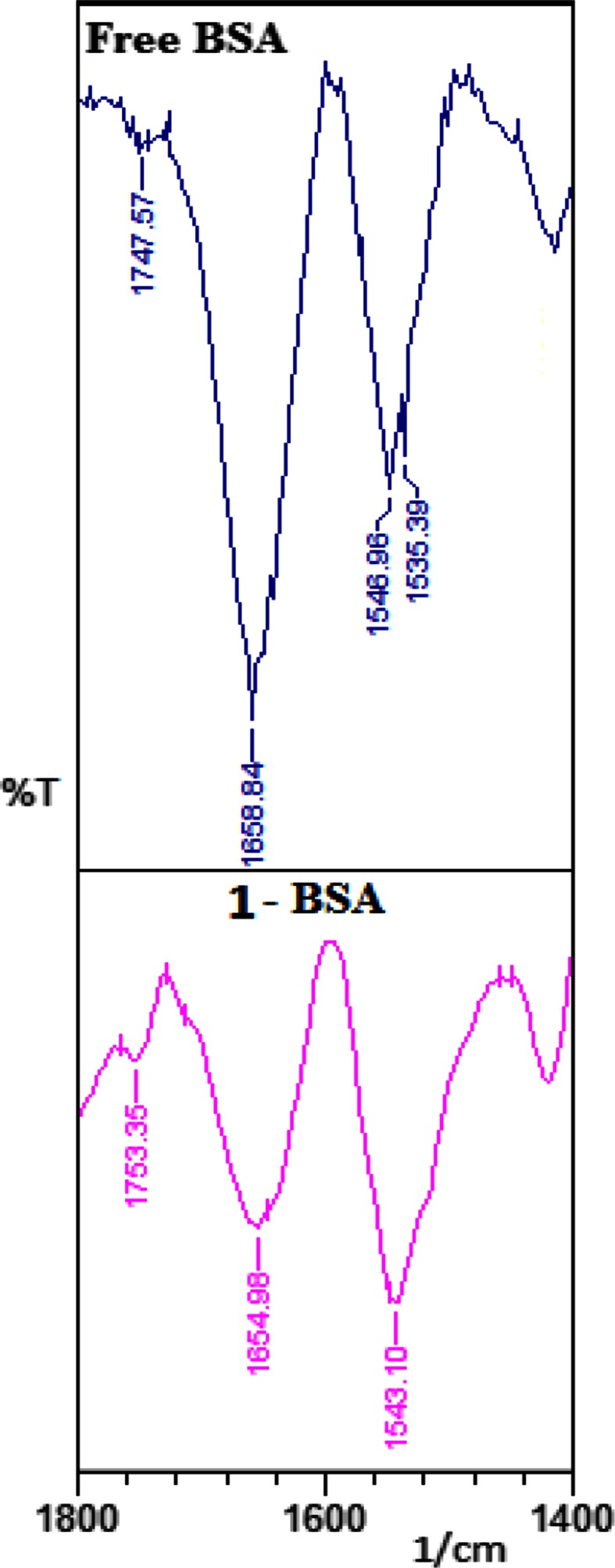
FTIR spectrum of free BSA and **1**-BSA

Shifting of protein amide I band (mainly C=O stretch) and amide II band (C=N stretching coupled with N–H bending modes) which originally absorbs at 1653 and 1541 cm^−1^, respectively (Byler and Susi 1986; Krimm and Bandekar 1986) was observed as **1** interacted with BSA protein (Fig. 5). In recent studies, the observed FTIR peaks for the free protein are reported to be: α-helix (1656 cm^−1^), β-sheet (1618 and 1628 cm^−1^), turn structure (1670 cm^−1^), β-antiparallel (1693 and 1680 cm^−1^) and random coil (1638 cm^−1^) (Ahmed-Ouameur et al. 2006; Bourassa et al. 2010). The β-sheet structure is composed of two components at 1618 (inter β-strand) and 1628 cm^−1^ (intra β-strand) (hydrated) that are consistent with the spectroscopic current studies of bovine serum albumin (Ahmed-Ouameur et al. 2006; Bourassa et al. 2010; Byler and Susi 1986; Krimm and Bandekar 1986).

On addition of **1** (0.00125 mM) to BSA, decrease in intensity as well as shift (4 cm^−1^) of the amide I band at 1658 cm^−1^ (BSA) was observed at 1654 cm^−1^. In the spectrum of **1**-BSA complex, the reduction in intensity due to loss of protein structure of the amide I band was obtained and suggests major reduction in protein α-helical structure (Fig. 5) (Ahmed-Ouameur et al. 2006; Bourassa et al. 2010). The decrease in α-helix structure and increase in β-sheet and turn structures is indicative of protein destabilization upon interaction with **1**.

On quantitative analysis of BSA and **1**, free protein was found to have 69 % a-helix (1658 cm^−1^), 12 % β-sheet (1620 cm^−1^), 14 % turn structure (1676 cm^−1^), 3 % β-antiparallel (1690 cm^−1^) and 2 % random coil (1637 cm^−1^) structure (Fig. 5). The results are consistent with the spectroscopic studies of bovine serum albumin previously reported (Ahmed-Ouameur et al. 2006; Bourassa et al. 2010). Upon interaction of **1**, a major decrease in α-helix from 69 % (free BSA) to 7.4 % (**1**-BSA at 0.00125 mM) was observed and 14 % in β-sheet (**1**-BSA, 0.00125 mM), 3.7 % in β-antiparallel (**1**-BSA, 0.00125 mM) and 4.7 % in random coil (**1**-BSA, 0.00125 mM) were obtained.

### Computational study

#### Huckel charge densities and dipole–dipole interactions

Often in organic chemistry, the “partial” charge on an atom of a compound highlights the nucleophilic or electrophilic site. In the current study, Huckel charge densities were analyzed theoretically as tabulated in Table 3. From this analysis, one thing is clear that P atom was obtained to have maximum positive charge (1.857) and was found to have better site for nucleophilic attack and double-bonded oxygen attached to P is itself may act like a nucleophile with a negative charge 1.184. Huckel charge densities are useful quantities to illustrate the charge distributions which can give the information about how the molecules interact with another molecule in environmental compartments.

**Table 3:** Optimized Huckel charges and geometric parameter for **1**

Huckel charges	Bond length (Å)	Bond angle (°)	Bond angle (°)
*Geometric* *parameters* *of* *product* *including* *Huckel* *charges*			
C −0.176 [C(1)] C −0.024 [C(2)] C −0.159 [C(3)] C −0.032 [C(4)] C −0.144 [C(5)] C 0.135 [C(6)] N 0.139 [N(7)] N −0.275 [N(8)] P 1.857 [P(9)] O −1.184 [O(10)] S −0.132 [S(11)] O −0.484 [O(12)] C −0.164 [C(13)] C 0.064 [C(14)] H 0.032 [H(15)] H 0.028 [H(16)] H 0.029 [H(17)] H 0.029 [H(18)] H 0.028 [H(19)] H 0.092 [H(20)] H 0.102 [H(21)] H 0.048 [H(22)] H 0.042 [H(23)] H 0.047 [H(24)] H 0.036 [H(25)] H 0.034 [H(26)] H 0.033 [H(27)]	C(1)–C(2) 1.387 C(6)–C(1) 1.387 C(1)–H(15) 1.067 C(2)–C(3) 1.380 C(2)–H(16) 1.074 C(3)–C(4) 1.387 C(3)–H(17) 1.070 C(4)–C(5) 1.378 C(4)–H(18) 1.072 C(5)–C(6) 1.393 C(5)–H(19) 1.070 C(14)–H(27) 1.083 C(14)–H(26) 1.079 C(14)–H(25) 1.078 C(13)–H(24) 1.078 C(13)–H(23) 1.077 C(13)–H(22) 1.077 O(12)–C(14) 1.450 S(11)–C(13) 1.890 P(9)–O(12) 1.637 P(9)–S(11) 2.225 P(9)–O(10) 1.536 N(8)–H(21) 1.010 P(9)–N(8) 1.711 N(7)–H(20) 1.007 N(7)–N(8) 1.430 C(6)–N(7) 1.418	H(27)–C(14)–H(26) 109.490 H(27)–C(14)–H(25) 110.562 H(27)–C(14)–O(12) 109.979 H(26)–C(14)–H(25) 110.378 H(26)–C(14)–O(12) 110.125 H(25)–C(14)–O(12) 106.264 H(24)–C(13)–H(23) 112.011 H(24)–C(13)–H(22) 110.472 H(24)–C(13)–S(11) 108.533 H(23)–C(13)–H(22) 110.981 H(23)–C(13)–S(11) 108.500 H(22)–C(13)–S(11) 106.117 Lp(30)–O(12)–Lp(29) 90.850 Lp(30)–O(12)–C(14) 109.500 Lp(30)–O(12)–P(9) 109.500 Lp(29)–O(12)–C(14) 109.500 Lp(29)–O(12)–P(9) 109.500 C(14)–O(12)–P(9) 123.201 C(13)–S(11)–P(9) 102.800 O(12)–P(9)–S(11) 106.677 O(12)–P(9)–O(10) 115.060 O(12)–P(9)–N(8) 96.629 S(11)–P(9)–O(10) 112.080 S(11)–P(9)–N(8) 108.756 O(10)–P(9)–N(8) 116.293 Lp(28)–N(8)–H(21) 90.360 Lp(28)–N(8)–P(9) 109.500	Lp(28)–N(8)–N(7) 109.500 H(21)–N(8)–P(9) 114.031 H(21)–N(8)–N(7) 114.741 P(9)–N(8)–N(7) 115.639 H(20)–N(7)–N(8) 111.651 H(20)–N(7)–C(6) 115.282 N(8)–N(7)–C(6) 115.324 C(4)–C(5)–H(19) 119.430 C(4)–C(5)–C(6) 120.640 H(19)–C(5)–C(6) 119.929 C(1)–C(6)–N(7) 122.839 C(1)–C(6)–C(5) 118.510 N(7)–C(6)–C(5) 118.627 C(3)–C(4)–H(18) 120.556 C(3)–C(4)–C(5) 120.774 H(18)–C(4)–C(5) 118.669 C(2)–C(3)–H(17) 120.633 C(2)–C(3)–C(4) 118.739 H(17)–C(3)–C(4) 120.626 C(1)–C(2)–H(16) 118.773 C(1)–C(2)–C(3) 120.830 H(16)–C(2)–C(3) 120.391 C(2)–C(1)–H(15) 118.950 C(2)–C(1)–C(6) 120.494 H(15)–C(1)–C(6) 120.554

The optimized value of dipole–dipole interactions for the molecule **1** is 0.12, which is low, and it conforms that molecule **1** could interact or bind less with other living or organic molecules such as proteins, enzymes, DNA; consequently, molecule **1** may cause low harm to living organisms and total environment. Biggest impact of these interactions on living organisms was seen in past with protein folding (Campbell and Reece 2005; Le-Fèvre 1953). Every process of protein binding depends on net dipole–dipole interactions (Campbell and Reece 2005; Le-Fèvre 1953). From the analysis of these parameters of molecule **1**, it is clear that this molecule may interact with the proteins, enzymes, DNA and essential organic molecules with low binding constant as was proved by low value of **1**-BSA interactions.

#### Geometric parameters and vibrational frequencies

Bond length and bond angles of **1** have been optimized (Table 3). These parameters may help the future studies like single-crystal analysis of **1**. The optimized theoretical vibrational frequencies of product were found to be in good agreement with the corresponding experimental data (Table 4). The slight differences observed between the calculated and experimental values were observed mostly due to fact that the theoretical calculations were performed for the product in the gaseous phase, while the experimental results were obtained for the solid phase of the pesticides (Young 2001).

**Table 4:** Optimized and experimental FTIR frequencies for **1**

Optimized frequencies (cm^−1^)^a^	Experimental frequencies^a^	Mode assignments	Shift values (cm^−1^)
3650	3640	*v*(O–H)	10
3350	3360	*v*(N–H)	10
1790	1890	*v*(N=N)	100
1671	1500	*v*(C=C)	121
980	1050	*v*(C–N), *v*(C–O), *v*(P=O)	70
835	830	*v*(P–O)	05
707	700	*v*(N–H) oop, monosub.	07

^a^Optimized frequencies are for gas phase, while experimental frequencies are in solid phase

#### Computational evaluation of structural analogs of **1** for physical and molecular properties

**Fig. 6:**
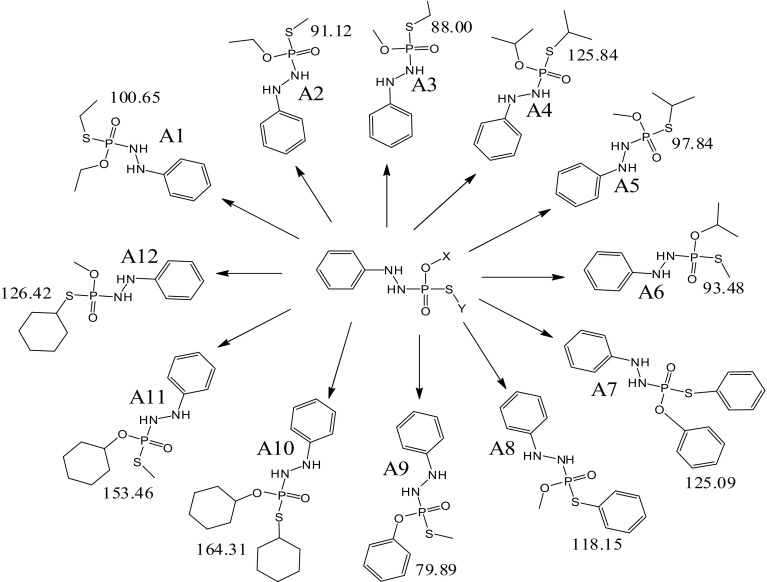
Chemical structures of new derivatives of **1** and values of average polarizability 〈*α*〉 in eV

Twelve new analogs of **1** were prepared computationally (Fig. 6) and compared with **1**. The HOMO–LUMO energy difference of analogs A1–A12 was calculated (Fig. 6; Table 5). It was obtained that all the molecules are non reactive due to their high energy difference. As per literature, the molecules with reduced HOMO–LUMO gaps <1.30 eV are chemically very reactive due to HOMO contribution to the decrease in the topological resonance energy (Aihara 1999). The observed value of energy difference for molecules A1–A12 lies between 33.311 and 1.542 eV. Hence, on the basis of HOMO–LUMO energy gap, the order of stability was: A11 ≫ A ≫ A9 = A6 > A10 > A8 > **1** = A2 = A3 > A1 > A4 > A12 > A5 ≫ > A7. But an interesting point was analyzed, i.e., once the phenyl molecule attached to **1,** it converted into least stable molecule A7 with energy gap 1.542 eV. Disturbance of planarity and polarity may a reason behind it, because N–N site potentially making the **1** more planar. The P atom of **1** is exactly the site that can be polarized, especially because N–N group, O–X group and S–Y groups attached to P are unequal.

**Table 5:** Statistical parameters of electronic and physicochemical properties of analogs of **1**

Code	Analog		Statistics		Physicochemical			Electronic		
	*X*	*Y*	Series	*R* ^2^	*E*_Steric_ (kcal/mol)	〈*α*〉	Dipole (D)	*E*_LUMO_ (eV)	*E*_HOMO_ (eV)	∆*E* (eV)
**1**	CH_3_	CH_3_			23.36	94.68	2.88	0.490	−6.319	6.809
A1	C_2_H_5_	C_2_H_5_	1^a^	0.98	25.38	100.65	4.55	0.490	−6.316	6.806
A2	C_2_H_5_	CH_3_			23.10	91.12	4.99	0.490	−6.319	6.809
A3	CH_3_	C_2_H_5_			23.98	88.00	5.79	0.490	−6.319	6.809
A4	C_3_H_9_	C_3_H_9_	2^a^	0.95	29.90	125.84	6.16	0.490	−6.310	6.800
A5	CH_3_	C_3_H_9_			24.56	97.84	6.63	0.490	−6.307	6.797
A6	C_3_H_9_	CH_3_			29.04	93.48	5.99	0.490	−6.353	6.843
A7	C_6_H_6_	C_6_H_6_	3^a^	0.93	21.62	125.09	6.13	0.490	−1.052	1.542
A8	CH_3_	C_6_H_6_			20.59	118.15	5.20	0.490	−6.321	6.811
A9	C_6_H_6_	CH_3_			23.57	79.89	5.04	0.490	−6.353	6.843
A10	C_6_H_12_	C_6_H_12_	4^a^	0.96	38.84	164.31	6.01	0.490	−6.351	6.841
A11	C_6_H_12_	CH_3_			32.87	153.46	2.27	26.97	−6.341	33.311
A12	CH_3_	C_6_H_12_			29.29	126.42	5.71	0.490	−6.309	6.799
A	–	–			20.19	55.57	4.30	2.313	−10.01	12.323

^a^Series 1,2,3 and 4 include acephate (A)

##### Polarizabilities and the prediction of the biodegradation

The computed averaged static dipole polarizability and dipole moment of molecules A1–A12 including **1** and acephate (A) were calculated. The component of the polarizability tensor was optimized along *z*-axis, i.e., along molecular axis. The values of average polarizabilities depend upon the positions of substituents bound to the moiety (Long 1982). The average polarizability (〈*α*〉) was calculated as per formula reported in the literature (Ostojic et al. 2014), i.e., 〈*α*〉 = (*α*_xx_ + *α*_yy_ + *α*_zz_)/3. The values of dipole moment and average polarizability for all molecules are tabulated in Table 5. As per recent computational studies, the biodegradation is directly proportional to the average polarizability of molecule (Long 1982; Ostojic et al. 2014). On the basis of average polarizability, the order of biodecomposition is: A9 > A3 > A2 > A6 > **1** > A5 > A1 > A8 > A7 > A4 > A4 > A12 > A11 > A10.

**Fig. 7:**
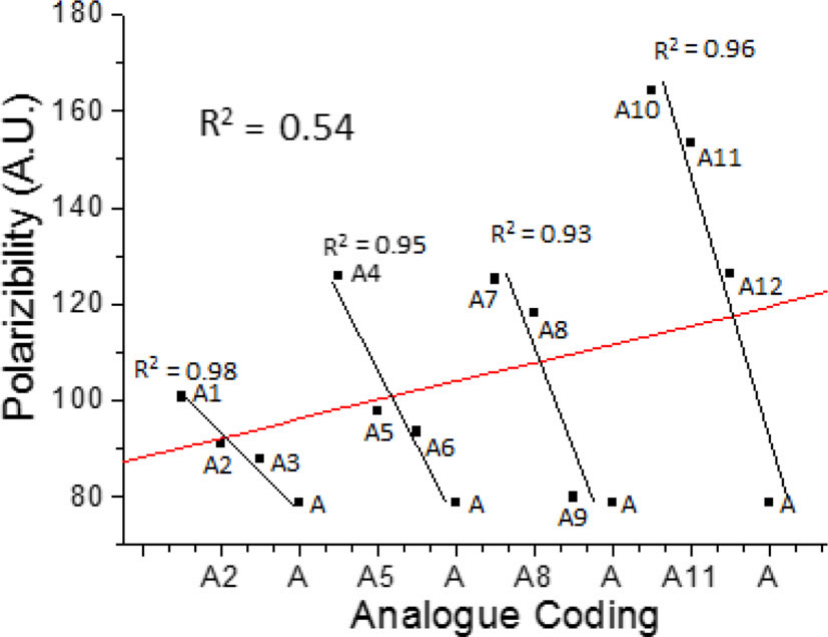
Relationship with different derivatives of **1**

A statistical relationship between polarizability of all the analogs was analyzed using origin 6.1 software. On the basis of substituents, analogs were divided into four series 1–4. Variations in polarizability of analogs were compared in all series w.r.t. acephate. Overall value of *R* ^2^ was 0.54, indicating that all the analogs having significant (*p* < 0.05) difference in polarizability. The *R* ^2^ value for series 1 (A, A1–A3) was 0.98, for series 2 (A, A4–A6) was 0.95, for series 3 (A, A7–A9) was 0.93 and for series 4 (A, A10–A12) was 0.96 (Fig. 7). Among the four series, order of biodecomposition was found in order 1 > 3 > 2 > 4.

## Conclusion

Phenylhydrazine binds with acephate through nucleophilic attack on it. The obtained product neutralized toxic effect of acephate on plant growth-promoting strains. The binding of **1** to BSA protein and interactions with PGPR strains may not be expected to explain the toxicity directly, but it can serve as a model to study the effect of **1** and other pesticides on proteins and microorganisms. A combined experimental and computational study was developed with the aim of evaluating and understanding the structural, energetic and stability of **1**.

## Safety

Acephate is an organophosphate pesticide that inhibits the activity of cholinesterase. Direct contact with this should be avoided. Work performed with this pesticide in the open should take place in a fume hood using gloves and eye protection.

## Electronic supplementary material

Below is the link to the electronic supplementary material.

Supplementary material 1 (DOC 875 kb)
