# Supplementary material for: Unexpected formation of N′-phenyl-thiophosphorohydrazidic acid O,S-dimethyl ester from acephate: chemical, biotechnical and computational study
Source: 3 Biotech. 2015 Dec 23;6(1):1. doi: 10.1007/s13205-015-0313-6 (PMC4689696; doi:10.1007/s13205-015-0313-6)
Supplement: Supplementary file 1 — Supplementary material 1 (DOC 875 kb) [file 13205_2015_313_MOESM1_ESM.doc]

**Supplementary Data**

**Unexpectedly formation of N'-Phenyl-thiophosphorohydrazidic acid O,S-dimethyl ester from acephate: Chemical, biotechnical and computational study**

Vijay Kumar^1^, Sukhmanpreet Kaur^1^, Simranjeet Singh^2^, Niraj Upadhyay*^3^

1. Department of Chemistry Lovely Professional University, Punjab- INDIA.
2. Department of Biotechnology, Lovely Professional University, Punjab- INDIA
3. Department of Chemistry, Dr. Harisingh Gour University, Madhya Pradesh - India

*Corresponding author: Niraj Upadhyay. Email: *nivij.res@gmail.com*

**Supplementary Information S1: Chemical details of acephate**

| **Pesticide** | **CAS No** | **Molar Mass(**g/mol**)** | **Melting Point (**^0^C**)** | **Structure** |
| --- | --- | --- | --- | --- |
| Acephate  (Insecticide) | 30560-19-1 | 183 | 93±3 |  |

**Supplementary Information S2: Instruments and experimental conditions**

IR spectra were recorded on Shimadzu FTIR Spectrophotometer (4000 – 400 cm^-1^) by preparing pallets in dry (at 120^0^C) KBr. ^31^P-NMR spectra of the free ligands and their complexes were recorded on Brucker Avance 400 MHZ spectrophotometer using DMSO-d_6_ as solvent and TMS as an internal reference. All the mass spectra were recorded on Waters, QTOF Micromass (LC-MS) spectrophotometer. Thermogravimetric analysis (TG and DTG) was carried out in the temperature range from 25 to 800°C in a steam of nitrogen atmosphere by Shimadzu TG 50H thermal analyzer. The experimental conditions were: platinum crucible, nitrogen atmosphere with a 30 ml/min flow rate and a heating rate 10 °C/min.

**Supplementary Information S3: Mathematical detail for interaction of 1 with BSA**

It is assumed that the interaction between the ligand L (**1**) and the substrate S (BSA protein) is 1:1; for this reason, a single complex SL (1:1) is formed. It was also assumed that all the binding sites are independent and follow the Beer’s law. A wavelength is selected at which the molar absorptivities, ε_s_ (molar absorptivity of the substrate) and ε_S11_(molar absorptivity of the complex) are different. Then at total concentration S_t_ of the substrate, in the absence of ligand and the light path length is b= 1 cm, the absorbance of solution is: A_0_ = ε_S_bS_t_ ---------- (1)

In the presence of ligand at total concentration L_t_, the absorbance of a solution containing the same total substrate concentration is: A_L_= ε_S_b[S] + ε_L_b[L] + Δε_11_b[SL] ----------- (2)

Where [S] is the concentration of the uncomplexed substrate, [L] is the concentration of the uncomplexed ligand and [SL] is the concentration of the complex. and Δε_11_= ε_11 -_ ε_S_ - ε_L_ (ε_L_ molar absorptivity of the ligand). By measuring the solution absorbance against a reference containing ligand at the same total concentration L_t_, the measured absorbance becomes;

A = ε_S_bS_t_ + Δε_11_b[SL] ----------- (3)

Combining Eq. (3) with the stability constant definition; K_11_=[SL]/[S][L], gives:

ΔA = K_11_ Δε_11_b[S][L] --------------------------(4)

Where, ΔA =A-A_0_. From the mass balance expression S_t_=[S]+[SL], we get [S]=S_t_/(1 +K_11_[L]), Putting value of [S] in Eq.(4),

ΔA/b = S_t_K_11_Δε_11_[L]/ (1 +K_11_[L]) ------------------- (5).

Eq.(5) is the binding isotherm, which shows the hyperbolic dependence on free ligand concentration. But, inverse of equation (5), may give rise to a straight line equation:

b/ΔA = (1/ S_t_K_11_Δε_11_[L]) + (1/ S_t_Δε_11_) --------------- (6)

Thus the double reciprocal plot of 1/ΔA versus 1/[L] is linear and the binding constant can be estimated from the following equation: K_11_= intercept/slope and ε_11_ can be obtained from the equation, ε_11_ = Δε_11_ + ε_s_ + ε_L_, where, Δε_11_ is obtained from the relation Δε_11_ = Intercept × S_t_

**Supplementary Information S4: Details of Thermal Analysis**

Acephate showed decomposition in two steps. The first decomposition step was accompanied within the temperature range of 40–300^0^C, by weight loss (Obs. = 50.06%, Calc. = 50.02%) which assigned to the loss of CH_3_SH and CH_3_COOH molecules. The second step showed loss in weight within the temperature range of 310–740^0^C, which is due to removal of CH_3_PO_2_N molecule, by weight loss (Obs. = 49.78%, Calc. = 49.82%). It was observed that entire acephate molecule decomposed below 620°C. The TG/DTG curve indicated that the decomposition process of acephate included two steps and the main decomposition occurred in the first step with temperature range from 420K to 470K and over 50% mass was degraded. The temperature of 460 K was the critical point where maximum mass loss occurred in the pyrolysis process of acephate. The main degradation compositions were acetaldehyde and methanethiol in this step. But second step was also equally contributing in the decomposition process. Second step was mainly lies form 670K to 920K and over 50% mass was degraded. The main degradation compositions were methanol, ammonia and phosphate-oxides.

Thermal analysis (TG/DTG) was used to get information about the thermal stability of the final product **1** as well as to verify the status of water molecules associated with product. The product was subjected to a TG/DTG analysis from ambient temperature to 800^0^C under nitrogen atmosphere with heating rate 10^0^C/min (Figure 5). Mass loss obtained from TG curves was in a good agreement with the calculated values. Product undergoes decomposition in two steps. The first decomposition step is accompanied within the temperature range of 40–120^0^C, by weight loss (Obs. = 5.68%, Calc. = 5.72%) which assigned to the loss of one CH_3_SH molecule. The second step shows loss in weight within the temperature range of 270–710^0^C, which is due to removal of one water molecule, one acephate molecule and one CH_3_SH molecule, by weight loss (Obs. = 28.88%, Calc. = 29.02%). It was observed that the final product remain stable after the 870°C, the remaining product (obs. = 51.63%, calcd. = 51.59%) contained phenylhydrazine, thiophosphorous acid O, S-dimethyl ester and acephate molecules.

**Supplementary Information S5: Plant growth promoting activities**

*Siderophoric activity analysis:* Bacterial cultures were inoculated into the King’s B media with and without (50 mg /L) presence of FeCl_3_ and incubated at 28 ± 2°C for 48hrs. The fluorescence pigment of the bacterial colonies and that diffuses in the surrounding agar were assessed using an ultraviolet lamp. Fluorescence pigment formed were considered as an indication of siderophore production.

*Phosphate solubilization analysis:*The ability of isolates to solubilize phosphate were evaluated qualitatively using Potato-Dextrose Yeast Extract Agar. Each bacterial culture were spot inoculated in the centre of the plate and incubated at 28±2°C for 10 days. Phosphate solubilization was assessed by measuring the clear/ halo zone. The halo zone was calculated by subtracting bacterial colony diameter from the total halo zone diameter.

*Hydrogen cyanide production analysis:* Isolates sub cultured on nutrient agar medium were supplemented with glycine (4/4 gL^-1^). The production of cyanide was detected after 48hrs from inoculation, using picrate/Na_2_CO_3_ paper fixed to the underside of the petri-dish lids which were scaled with parafilm before incubation at 28°C. A change from yellow to orange, red, brown, or reddish brown was recorded as an indication of weak, moderate, or strongly cyanogenic potential, respectively.

*Indole acetic acid analysis:* Nutrient broth (50 ml) containing DL-Tryptophan was inoculated with 500 µL of 24 hrs old bacterial cultures and incubated in refrigerated incubator shaker at 30±0.1°C and 180 rpm for 48 hrs in dark. The bacterial cultures were centrifuged at 10,000 rpm for 10 minute at 4^0^C. Estimation of Indole acetic acid in the supernatants was done using colorimetric assay. One millimeter of supernatant was mixed with Salkowski reagent and absorbance of the resultant pink color was calculated after 535nm in UV-vis spectrophotometer. Appearance of pink color in test tubes indicates IAA production.

*Statistical analysis:* The experiments were conducted in three replicates using the same treatments. The difference among treatment means was compared by high range statistical domain using ANOVA test at (p ≤0.05) level.


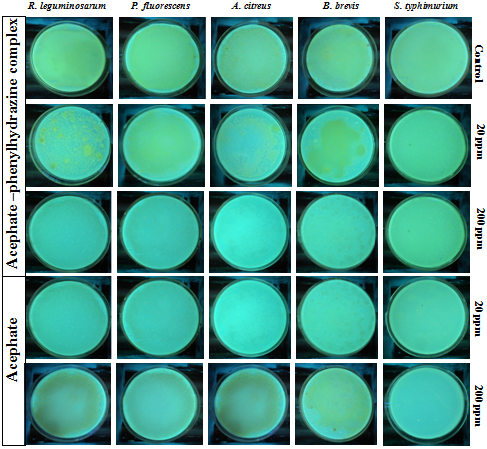


**Figure S2:** Comparative siderophore production images of acephate and product at 20 and 200 ppm.


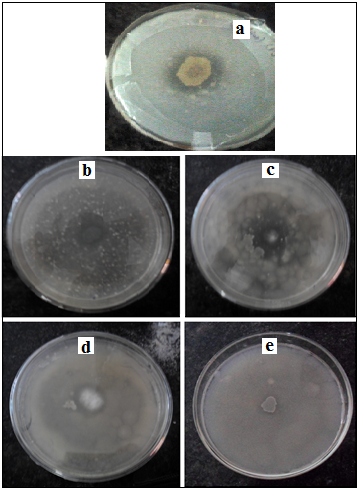


**Figure S3:** Comparative phosphate solubilization images with *Pseudomonas fluorescens* strain; control (a) product (b & c) and acephate (d & e) at 20 and 200ppm.


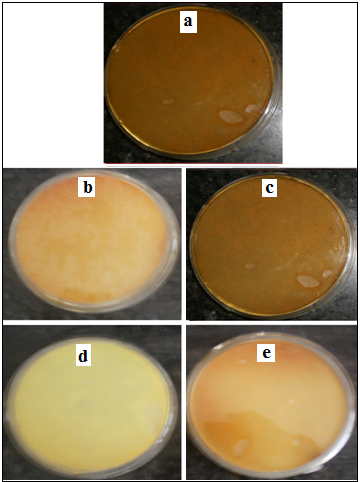


**Figure S4:** Comparative hydrogen production images with *Pseudomonas fluorescens* strain; control (a) product (b & c) and acephate (d & e) at 200 and 20ppm.


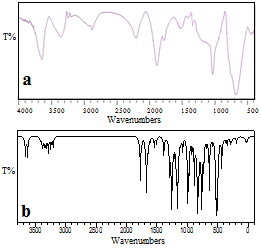


**Figure S5:** Comparative FTIR spectra of product; experimental (a) and optimized (b).


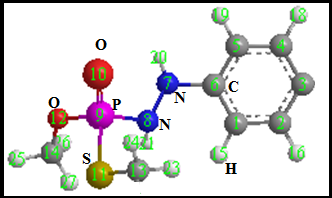


**Figure S6:** Labelled figure of molecule 1.
